# World’s Congress Transactions

**Published:** 1893-10

**Authors:** Pemberton Dudley

**Affiliations:** General Secretary A. I. H.


### WORLD'S CONGRESS TRANSACTIONS.

Editor Homœopathic Physician :—The American Institute of Homœopathy at its recent meeting authorized the Executive Committee to confer with the officials of the World's Congress, with power to act in reference to the publication of the Congress proceedings. I have now to report that the manuscripts were placed in the hands of the General Secretary on August 18th, and under the direction of the Committee will be issued in a separate volume.

All Institute members not in arrears, and all foreign physicians who contributed in any way to the success of the Congress will receive a copy free. Others may obtain copies by sending five dollars to the Treasurer, Dr. T. F. Smith, 264 Lenox Avenue, New York city.

PEMBERTON DUDLEY,
*General Secretary A. I. H.*

